# Molecular Epidemiology and Genotyping of *Blastocystis* sp. Among Patients in Guilan, Northern Iran

**DOI:** 10.3390/tropicalmed11080227

**Published:** 2026-08-12

**Authors:** Mohammad Reza Mahmoudi, Zahra Naemi, Meysam Sharifdini, Nozhat Zebardast, Somayeh Abbaszadeh, Panagiotis Karanis

**Affiliations:** 1 Biomedicine Research Center, Trauma Institute, Guilan University of Medical Sciences, Rasht P.O. Box 3363, Iran; 2 Department of Parasitology, School of Medicine, Guilan University of Medical Sciences, Rasht P.O. Box 3363, Iran; zahranaemi3176@gmail.com (Z.N.); sharifdini5@gmail.com (M.S.); yegane20171396@gmail.com (S.A.); 3 Cellular and Molecular Research Center, School of Medicine, Guilan University of Medical Sciences, Rasht P.O. Box 3363, Iran; nszebardast@gmail.com; 4 Medical Faculty and University Hospital, University of Cologne, P.O. Box 57931 Cologne, Germany; 5 University of Nicosia Medical School, P.O. Box 24005, Nicosia CY-1700, Cyprus

**Keywords:** *Blastocystis* sp., subtypes, Iran, molecular epidemiology

## Abstract

Background: *Blastocystis* sp. is an anaerobic intestinal protozoan with extensive genetic diversity and controversial pathogenicity. The distribution and molecular subtypes of this entity in Guilan province, northern Iran, remain under-investigated. Objective: This study aimed to determine the prevalence and molecular subtypes of *Blastocystis* sp. in Rasht, Guilan Province, Iran. Methods: In this cross-sectional study (2023–2024), 300 stool samples were collected from patients referred to the medical laboratory at Razi Hospital. Microscopic examination and PCR amplification targeting the SSU rDNA gene were performed. Sequencing was performed on the positive isolates, and subtyping was achieved through BLAST alignment and subsequent phylogenetic analysis. Results: The overall prevalence of *Blastocystis* sp. was 12.3% (*n* = 37). No significant associations were found between infection and age, sex, or place of residence (*p* > 0.05). Molecular characterisation (23.08%). No statistically significant correlation was observed between subtypes and clinical symptoms, although ST1 and ST7 were more frequently detected in symptomatic individuals. Conclusions: *Blastocystis* sp. showed notable subtype diversity in the studied population, including the potentially zoonotic ST7. However, no significant association was found between subtypes and clinical manifestations. These findings support sustained molecular epidemiological surveillance and larger-scale studies to clarify subtype-specific pathogenesis and transmission dynamics.

## 1. Introduction

*Blastocystis* sp. is a widely distributed anaerobic protozoan that infects both humans and animals, with prevalence rates reported as high as 60% in developing nations and 5–20% in more industrialised regions. It is frequently isolated from stool samples in parasitology laboratories. Recent studies have highlighted the clinical relevance of this parasite in humans. Approximately one billion people worldwide have been found to harbour this microorganism, which appears in several distinct morphological forms in stool examinations [1]. *Blastocystis* sp. is primarily transmitted via the faecal–oral route, and its prevalence increases in settings with poor hygiene and frequent animal contact [2]. *Blastocystis* sp. has been detected in both symptomatic and asymptomatic individuals, suggesting a possible, but not yet fully understood clinical relevance [3]. Despite extensive research, knowledge of this organism’s pathogenesis remains limited. *Blastocystis* infection has been associated with non-specific gastrointestinal symptoms such as abdominal pain, diarrhoea, nausea, vomiting, bloating, and anorexia, as well as less frequent dermatological complaints like urticaria and severe itching [4]. Although the parasite is detected in both symptomatic and asymptomatic carriers, immunocompromised individuals, such as those with HIV/AIDS and cancer, are recognised as high-risk populations for *Blastocystis*-related diarrheal illness [5,6].

Although *Blastocystis* sp. was traditionally considered a pathogen, recent evidence highlights its interactions with the gut microbiome and its potential role as a commensal or beneficial modulator of gut health. These findings represent a paradigm shift in *Blastocystis* research, redirecting attention from presumed pathogenicity to its influence on gut microbial balance and overall health [7]. *Blastocystis* sp. has been shown to modulate the abundance of specific bacterial taxa and the Bacillota/Bacteroidota ratio. Notably, accumulating evidence indicates that its effects on the microbiota may be subtype-dependent. Recent studies have also investigated the organism’s capacity to influence host immune responses, as it can induce both pro-inflammatory and anti-inflammatory cytokines. Moreover, *Blastocystis* may exert immunomodulatory effects that contribute to attenuation of the host immune response. These microbial and metabolic perturbations may subsequently affect neurotransmitter and other signalling molecule production, thereby influencing brain function and behaviour.

In addition, *Blastocystis* shows marked genetic diversity, with at least 42 subtypes identified among different isolates based on polymorphisms in the small-subunit ribosomal RNA (SSU rRNA) gene. Although these subtypes share a core set of genes, they differ in several key genomic characteristics, including genome size, intron number, guanine–cytosine (GC) content, and gene composition [4].

*Blastocystis* has been historically understudied, often regarded as a potential pathogen due to its association with gastrointestinal symptoms in some individuals [8]. More than 90% of human-derived *Blastocystis* isolates have been classified as ST1 to ST4, based on epidemiological data. Although these four subtypes are also found in animals, they are predominantly transmitted from person to person. Several subtypes are either infrequently detected or rarely reported in humans and are more typically associated with animals such as mammals and birds. For instance, ST5 is prevalent in pigs, while ST6 and ST7 are primarily found in poultry. ST10 and ST14 are commonly isolated from cattle [9,10]. Investigations conducted in various parts of Iran have confirmed the presence of *Blastocystis* subtypes ST1, ST2, ST3, ST4, ST5, and ST7 in human samples [11,12,13,14,15]. The prevalence of *Blastocystis* sp. infection varies widely across geographical regions and diagnostic methods. However, current knowledge about the parasite’s distribution and its subtypes in northern Iran remains limited. Therefore, the current study was conducted to characterise the prevalence and subtypes of *Blastocystis* circulating in northern Iran.

## 2. Materials & Methods

### 2.1. Study Design

This cross-sectional descriptive study was conducted from March 2023 to February 2024 at Guilan University of Medical Sciences in Guilan Province, northern Iran. Three hundred stool samples were collected from patients referred to the medical laboratory at Razi Hospital, a major regional referral centre for patients with gastrointestinal symptoms. Patients of all ages and both sexes were included after obtaining informed consent.

### 2.2. Sample Collection and Microscopic Examination

Microscopic examination (wet mount) was primarily used as an initial screening tool to confirm the presence of *Blastocystis* before proceeding to the molecular stage. Fresh stool samples were collected in sterile, labelled containers and promptly transported to the Parasitology Laboratory at the Faculty of Medicine, Guilan University of Medical Sciences. Each sample was examined using direct wet mounts (normal saline or Lugol’s iodine) and the formalin-ether concentration method under a light microscope. Positive samples were stored at −20 °C for subsequent molecular analysis.

### 2.3. DNA Extraction and Molecular Identification

**Figure 1:**
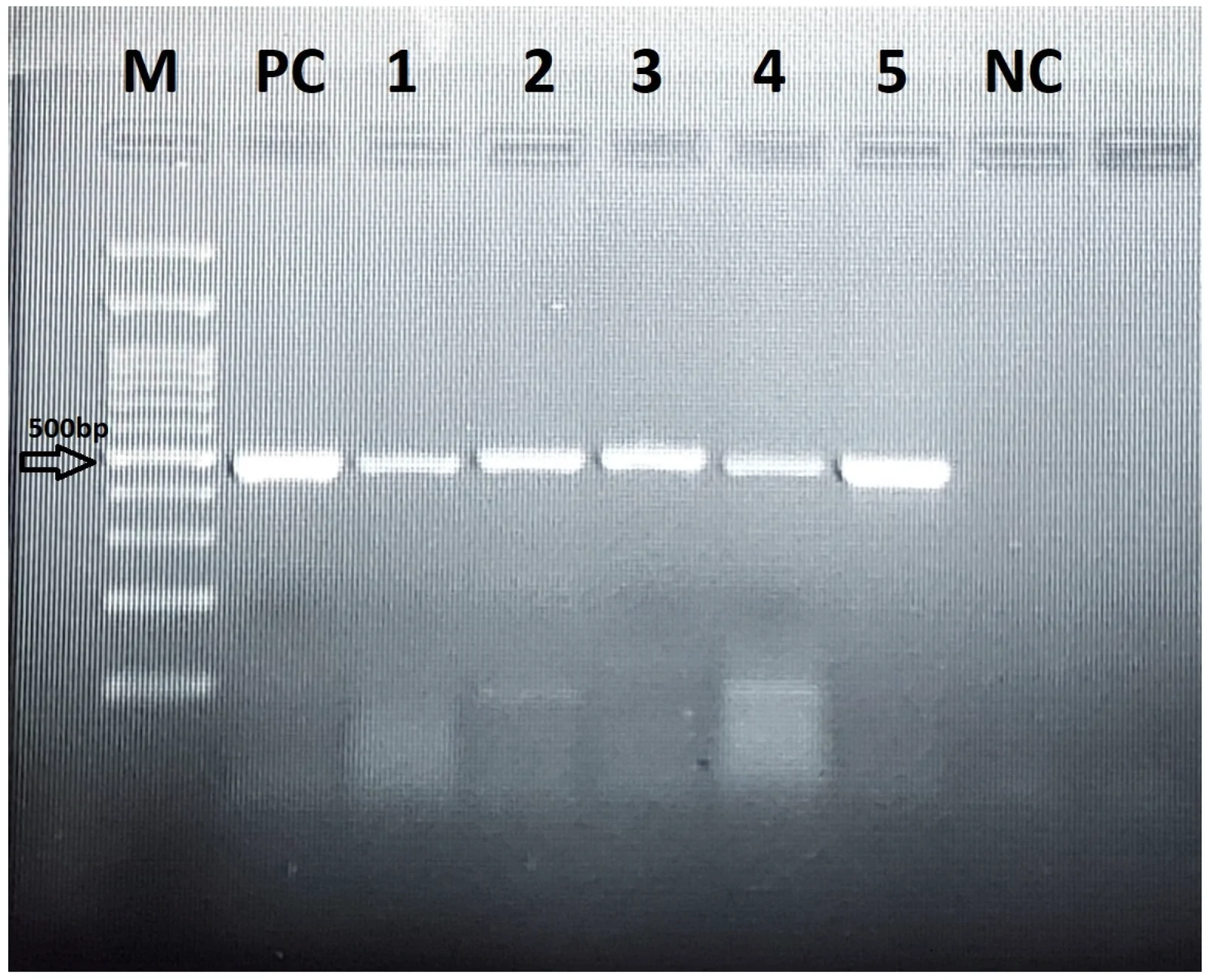
Visualisation of the amplification PCR products on agarose gel. M, 100 bp molecular weight marker; lanes 1–5, clinical *Blastocystis* isolates; PC, positive control; NC, negative control.

DNA of positive samples was extracted using a DNA Extraction Kit (Yakta Tajhiz, Tehran, Iran), according to the manufacturer’s instructions. The specific primers targeting a ~500 bp region used to amplify the SSU rDNA gene from *Blastocystis*-positive specimens were 505–532 (5′-GGA GGT AGT GAC AAT AAATC-3′; forward) and Blast 998–1017 (5′-TGC TTT CGC ACT TGT TCATC-3′; reverse). PCR reactions were set up in a final volume of 30 μL using Ampliqone Taq DNA Polymerase Master Mix RED (Odense, Denmark). Each reaction mixture contained 25 μL of the master mix, approximately 10 ng of template DNA, 0.1 μM of each primer, and distilled water to achieve the final volume. The thermal cycling protocol consisted of an initial denaturation at 94 °C for 3 min, followed by 33 cycles of denaturation at 95 °C for 35 s, annealing at 56 °C for 45 s, and extension at 72 °C for 1 min. A final elongation step was performed at 72 °C for 5 min (Figure 1).

### 2.4. Analysis Sequencing

**Figure 2:**
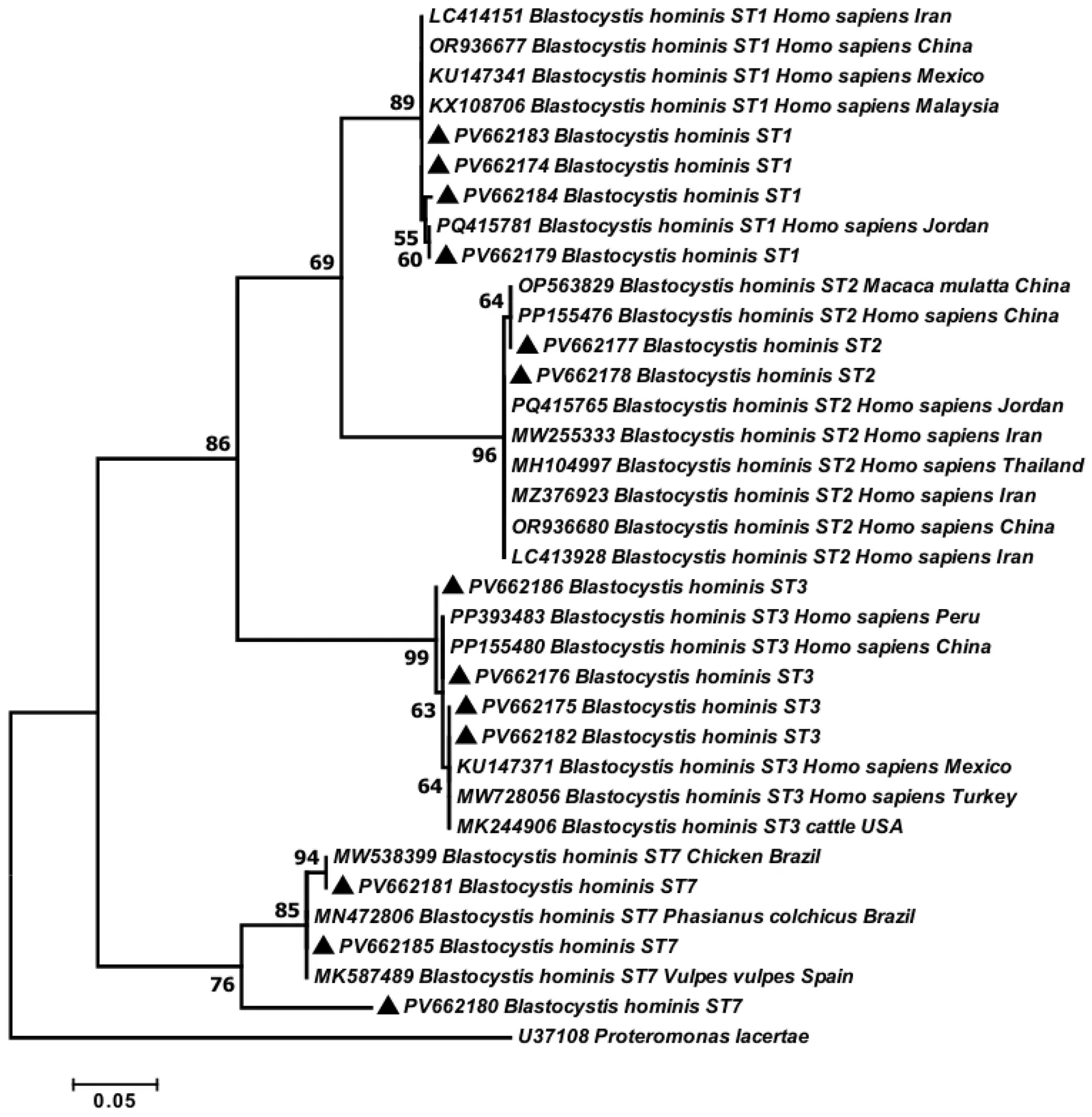
Phylogenetic tree of isolates of *Blastocystis* sp. obtained in the current study (▲) and reference sequences from previous studies based on SSU rRNA nucleotide sequences and constructed using the Tamura-Nei model in MEGA software version 6.

Due to funding limitations, 13 high-quality PCR products were randomly selected from the available products and sent to Codon Company for sequencing. Forward primers were used, and sequence analysis was performed in one direction. Sequence results were edited and analysed using Chromas version 2.01 (Technelysium Pty Ltd., Brisbane, Queensland, Australia) and compared to the GenBank database using the BLAST programs (http://www.ncbi.nlm.nih.gov/, accessed on 16 July 2026). Phylogenetic trees were constructed using Maximum-Likelihood algorithm and Tamura-3-parameter model in the MEGA 6.0 software., and bootstrap values from 1000 replications were used to assess tree topology reliability (Figure 2).

### 2.5. Ethical Considerations

This study was conducted in accordance with the ethical principles outlined in the Declaration of Helsinki. The research protocol was reviewed and approved by the Research Council and Ethics Committee of Guilan University of Medical Sciences (Approval Code: IR.GUMS.REC.1402.471). Written informed consent was obtained from all participants or their legal guardians before sample collection. All personal data was kept confidential.

### 2.6. Statistical Analysis

Data were analysed using SPSS version 26.0 software (IBM Corp., Armonk, NY, USA). Demographic characteristics, including age, sex, place of residence, and clinical symptoms, were summarised using descriptive statistics (frequencies and percentages). The association between *Blastocystis* hominis infection and categorical variables, including sex, place of residence, and clinical symptoms, was assessed using the Chi-square (χ^2^) test. A *p*-value < 0.05 was considered statistically significant.

## 3. Results

Microscopic examination identified *Blastocystis* sp. in 37 of the 300 samples (12.3%). The prevalence of *Blastocystis* sp. was slightly higher among females (23/153; 7.67%) than males (14/147; 4.67%), but this difference was not statistically significant (χ^2^ = 2.104; *p* = 0.14). Distribution by age group showed the highest number of positive cases in the 20–30 and 30–40-year age groups; however, no significant association between age and infection was found (*p* = 0.073). Similarly, while urban residents accounted for 26 (8.67%) of the 37 positive cases and rural residents for 11 (3.67%), no significant association was found between *Blastocystis* infection and place of residence (*p* = 0.727) (Table 1). A total of 300 stool samples were collected and analysed, consisting of 147 males (49%) and 153 females (51%), with an age range from 2 to 97 years and a mean age of 45.04 years (SD: 16.4). The largest number of participants was in the 20–30-year age group (35.66%), followed by the 30–40-year age group (22.33%). Most individuals resided in urban areas (*n* = 218; 72.67%), while 82 (27.34%) were from rural areas. Comparisons of the sequences from isolates with other available reference sequences in GenBank, using the BLAST system, revealed the presence of ST1 (*n* = 4; 30.77%), ST2 (*n* = 2; 15.38%), ST3 (*n* = 4; 30.77%), and ST7 (*n* = 3; 23.08%). ST1 and ST3 were equally prevalent, followed by ST7.

All sequence data for *Blastocystis* nucleotides obtained in the current study were deposited in GenBank under accession numbers PV662174–PV662186 as follows: ST1 was represented by four samples (PV662174, PV662179, PV662183, and PV662184), ST3 by four samples (PV662175, PV662176, PV662182, and PV662186), ST2 by two samples (PV662177–PV662178), and ST7 by two samples (PV662181–PV662185). A phylogenetic tree was constructed from 13 nucleotide sequences: 12 from the current study and 1 GenBank reference sequence, with *Proteromonas* lacertae (U37108) as the outgroup. Four subtypes were distinguishable: ST1, ST2, ST3, and ST7 (Figure 1).

Subtype distribution by gender showed no statistically significant differences (*p* = 0.65), although ST2 was observed only in females. By residence, ST1 and ST3 were more common among urban participants, while ST7 was more frequent among rural participants (*p* = 0.205). By age, ST3 was mainly observed in individuals aged 20–40 years, whereas ST7 was observed in older age groups; however, no significant association was found between age group and subtype (*p* = 0.28). Among the 37 individuals infected with *Blastocystis* sp., 18 (48.65%) reported gastrointestinal symptoms, including abdominal pain, nausea, and intestinal inflammation. Notably, 10 individuals (27.03%) were completely asymptomatic. No statistically significant correlation was found between symptoms and subtypes (*p* = 0.15). Symptomatic cases were mainly associated with ST1 (*n* = 3) and ST7 (*n* = 2), while all ST3 carriers and one ST2 carrier were asymptomatic. Finally, age comparisons between positive and negative individuals showed no significant difference (mean age: 45.49 vs. 42.57 years; *p* = 0.35, Mann–Whitney U test). Subtype-specific age comparisons using the Kruskal–Wallis test also revealed no statistically significant differences (*p* = 0.202). However, ST7 cases were observed in the youngest individuals, and ST3 carriers tended to be older (Table 2).

## 4. Discussion

In this study, the overall prevalence of *Blastocystis* sp. detected by microscopic examination was 12.3%, which aligns well with earlier reports from various provinces in Iran [5,13,14,15]. In contrast to the present study, Badparva et al. reported an overall prevalence of *Blastocystis* sp. of 3% in Iran between 2003 and 2015, with a decrease over this period [14]. Prevalence varies by geographic location, ranging from 60% in developing countries to 5–20% in developed countries [15,16]. Sampling size, diagnostic method, and hygiene may affect prevalence. Therefore, larger future studies may help clarify the trend of *Blastocystis* sp. in Guilan province.

Molecular studies on *Blastocystis* sp. conducted in Iran previously identified ST1, ST2, ST3, ST4, ST5, ST6, and ST7 in humans, and ST3 was reported in all studies conducted in Iran [1,5,13,17,18,19,20,21,22]. In this study, the predominance of ST3 and ST1 is consistent with previous molecular epidemiological studies conducted in Italy [23]. Generally, subtypes 3 has been identified as the most common subtype in human [24]. Subtype 3 was the most prevalent *Blastocystis* subtype in several studies of human samples from Iran [1,5,13,17,18,19], whereas two studies reported a higher incidence of subtype 1 in humans [5,18]. Khoshnood et al. reported that ST4 was the most prevalent subtype in human samples [1]. ST2 and ST7 were also identified in our study, and ST7 remains low compared with other subtypes reported in Iran and other countries [1,14].

Variations in *Blastocystis* subtype prevalence may reflect differences in lifestyle and animal reservoirs across study areas. In Guilan Province, high humidity and substantial rainfall may favour subtypes that persist in moist environments. Intensive agricultural practices, particularly rice cultivation and irrigation-based farming, may influence transmission dynamics and promote the spread of waterborne subtypes. These differences suggest the need for further investigation through expanded sampling and sequencing of isolates.

In our study, ST1 showed the highest frequency among individuals presenting with gastrointestinal symptoms. Several studies have suggested that ST1 is more often associated with clinical symptoms, whereas ST2 is more often observed in asymptomatic individuals. However, these associations may vary by region and host factors [16].

Distinct subtypes of *Blastocystis* sp. may differ in their effects on the gut microbiota and host health. Some analyses have shown that carriage is influenced by geography and lifestyle. ST1 and ST2 were more common in non-Westernized populations, whereas ST4 predominated in Europe [7]. In some studies, ST7 and ST3 have been associated with the pathogenesis of acute intestinal problems [8,25]. A study by Deng et al. showed that ST4 and ST7 infections exert strikingly differential effects on the gut microbiota, which could influence susceptibility to colitis [26]. Since ST7 is primarily found in poultry, detecting potentially pathogenic and zoonotic subtypes such as ST7 in the region suggests a transmission risk linked to local poultry farming practices and human–animal contact patterns.

The molecular characterisation of *Blastocystis* sp. in İzmir, Turkey, revealed ST3 as the most prevalent subtype (51.0%), followed by ST2 (34.5%) and ST1 (14.5%) [27]. Our study showed a different distribution: ST1 (30.77%), ST2 (15.38%), ST3 (30.77%), and ST7 (23.08%). The presence of ST1, ST2, and ST3 in both populations underscores their widespread circulation. As in the aforementioned study [28], we found no statistically significant association between specific subtypes and clinical manifestations. These findings suggest that pathogenicity may not be strictly genotype-dependent or that the sample size was insufficient to detect subtle subtype-symptom correlations. Larger studies and more extensive sequencing will be essential to clarify these relationships.

The identification of ST7 in our study, which was not reported in the aforementioned Turkish study [27], is of particular interest because it highlights the need for continuous molecular surveillance to monitor emerging subtypes in the region.

Statistical analysis showed no significant association between *Blastocystis* prevalence or its subtypes and age, sex, occupation, education level, place of residence, contact with animals, or clinical manifestations of infection in patients (*p* > 0.05) [29]. Salehi et al. reported a statistically significant association between gastrointestinal symptoms and *Blastocystis* infection in patients from southwestern Iran [30].

According to global surveys, *Blastocystis* sp. infection is common among schoolchildren, likely due to poor hygiene standards in this age group [28]. In the present study, the majority of positive cases were observed in the 20–30 age group, followed by the 30–40 age group. This may be because most participants in this study were in these two age groups, leading to more positive cases than in other age groups. No statistically significant correlation was observed between participants’ age, sex, residence, and the presence of *Blastocystis* infection.

The primary objective of this study was to assess the prevalence and genotypes of *Blastocystis* sp. in the study area. While the findings offer valuable insights, they also have certain limitations. Due to the limited number of positive samples and sequenced isolates, the results cannot be generalised to the entire Guilan population. Future comprehensive studies with larger sample sizes and sequencing of all positive samples are recommended to improve the reliability of these findings.

## 5. Study Strengths and Limitations

This study provides novel molecular epidemiological data on *Blastocystis* subtypes in Guilan province, contributing to the understanding of its genetic diversity in Iran. However, certain limitations should be acknowledged, including the moderate sample size, reliance on self-reported symptoms, and absence of quantitative assessments of parasite burden. Future studies with larger sample sizes, longitudinal designs, and advanced genomic tools are recommended to clarify the clinical significance of *Blastocystis* subtypes.

## 6. Conclusions

This molecular epidemiological study shows that *Blastocystis* sp. subtypes are diverse among patients in Rasht, northern Iran, with ST3 and ST1 being the most prevalent, followed by ST7 and ST2. The detection of potentially pathogenic and zoonotic subtypes, such as ST7 and ST1, emphasises the need for further studies to identify *Blastocystis* subtypes across different hosts and regions. These findings provide a basis for future investigations into the epidemiology, transmission dynamics, and public health impact of *Blastocystis* in Iran. However, they cannot be generalised beyond the study area. Further studies are required to determine the distribution of *Blastocystis* sp. subtypes within the population.

## Figures and Tables

**Table 1:** Prevalence of *Blastocystis* sp. infection according to age, sex, and place of residence.

Variables	No. of Examined	No. of Positive (%)	No. of Negative (%)	*p* Value
Age				
0–10	4	2 (0.67)	2 (0.67)	0.073
10–20	10	2 (0.67)	8 (2.67)	
20–30	107	10 (3.33)	97 (32.33)	
30–40	67	13 (4.33)	45 (18.00)	
40–50	46	4 (1.33)	42 (14.00)	
50–60	39	3 (1.00)	36 (12.00)	
60–70	22	2 (0.67)	20 (6.67)	
70–80	3	0 (0.00)	3 (1.33)	
80–90	2	1 (0.33)	1 (0.33)	
Sex				
Male	147	14 (4.67)	133 (44.33)	0.14
Female	153	23 (7.67)	130 (43.33)	
Residence				
Urban	218	26 (8.67)	192 (64.00)	0.727
Rural	82	11 (3.67)	71 (23.67)	

**Table 2:** Distribution of *Blastocystis* subtypes according to age, gender, residence, and clinical manifestations.

Variable	Variable	ST1	ST1	ST2	ST2	ST3	ST3	ST7	ST7	*p* Value
		Number	Percent	Number	Percent	Number	Percent	Number	Percent	
sex	Male	2	15.38	0	0.00	1	7.69	1	7.69	0.65
sex	Female	2	15.38	2	15.38	3	23.08	2	15.38	0.65
residence	Urban	3	23.08	1	7.69	4	30.77	0	0.00	0.052
	Rural	1	7.69	1	7.69	0	0.00	3	23.08	0.052
age	10–20	0	0.00	0	0.00	1	7.69	0	0.00	0.28
age	20–30	3	23.08	1	7.69	1	7.69	0	0.00	0.28
age	30–40	1	7.69	1	7.69	1	7.69	1	7.69	0.28
age	40–50	0	0.00	0	0.00	0	0.00	2	15.38	0.28
age	60–70	0	0.00	0	0.00	1	7.69	0	0.00	0.28
Clinical symptoms	Yes	3	23.8	1	7.69	0	0.00	2	15.38	0.15
Clinical symptoms	No	1	7.69	1	7.69	4	30.77	1	7.69	0.15

## Data Availability

The original contributions presented in this study are included in the article. Further inquiries can be directed to the corresponding authors.
